# Temperament and Character among mothers of individuals with gender dysphoria: a case-control study

**DOI:** 10.1192/j.eurpsy.2023.1336

**Published:** 2023-07-19

**Authors:** A. Talaei, S. Omidvar Tehrani, Z. Talaei

**Affiliations:** 1 Psychiatry and Behavioral Sciences Research Center, Mashhad University of Medical Sciences, Mashhad, Iran, Islamic Republic Of

## Abstract

**Introduction:**

Parents of individuals with gender dysphoria may experience distress when dealing with their child’s condition, and how they react can have a significant effect on their own as well as their child’s mental health.

**Objectives:**

In this study, we aimed to explore the personality traits among mothers of individuals with gender dysphoria in comparison to the mothers of individuals with cis-gender identity by utilizing the Temperament and Character Inventory (TCI) tool.

**Methods:**

We enrolled 27 mothers of GD individuals who had obtained licenses for gender affirmation surgery and 28 mothers of cisgender controls for this case-control study. Personality traits were measured by a validated Farsi version of the Temperament and Character Inventory (TCI) tool.

**Results:**

The mean±standard deviation age of mothers with GD children and controls was 53.95±9.44 and 53.00±7.28 years, respectively. 20 of the GD children were born with the female sex. Overall, TCI scores were statistically different between the two groups (p=0.03); however, this difference was only observed among Character scores (p=0.01) and was not significant in Temperament scores (p=0.33). We found significantly higher mean Cooperativeness (CO) and Self-Transcendence (ST) scores in the case group (p=0.007 and 0.031, respectively). We also identified significantly more individuals with a high CO score amongst mothers of GD individuals (Odds Ratio: 5.0, 95% Confidence Interval 1.2-21.0, p=0.028).

**Table:** 

**characteristics**	**Group**	**Mean**	**Std. Deviation**	**P-value**
**CO**	GD Control	20.2963 17.6786	2.28397 4.29516	**0.007**
**SD**	GD Control	16.7407 15.7500	3.49277 6.25167	0.474
**ST**	GD Control	9.7407 8.0000	2.98190 2.84149	**0.031**
**HA**	GD Control	7.7778 6.7143	4.50071 4.51218	0.386
**NS**	GD Control	7.0370 7.2143	2.99334 3.40324	0.839
**P**	GD Control	3.5926 3.2857	1.11835 2.07020	0.499
**RD**	GD Control	8.8519 7.7857	2.56760 1.93136	0.087

**Conclusions:**

By showing more mature, understanding, and kind personalities, the mothers of GD cases who have obtained licenses for gender affirmation surgery, have likely provided a positive atmosphere for the gender identity development and transition of their children. Additionally, their personalities were possibly better suited to deal with their child’s condition through having better compensatory adaptive traits.

**Disclosure of Interest:**

None Declared

